# Corrosion Behavior of Additively Manufactured GRX-810 Alloy in 3.5 wt.% NaCl

**DOI:** 10.3390/ma18143252

**Published:** 2025-07-10

**Authors:** Peter Omoniyi, Samuel Alfred, Kenneth Looby, Olu Bamiduro, Mehdi Amiri, Gbadebo Owolabi

**Affiliations:** 1 Department of Mechanical Engineering, Howard University, Washington, DC 20059, USA; 2 Department of Mechanical Engineering, George Mason University, Fairfax, VA 22030, USA; 3 Department of Mechanical Engineering Technology, Hagerstown Community College, Hagerstown, MD 21742, USA

**Keywords:** GRX 810, corrosion, high entropy alloy, HIP, oxide film, microstructure

## Abstract

This study examines the corrosion characteristics of GRX-810, a NiCoCr-based high entropy alloy, in a simulated marine environment represented by 3.5 wt.% NaCl solution. The research employs electrochemical and surface analysis techniques to evaluate the corrosion performance and protective mechanisms of this alloy. Electrochemical characterization was performed using potentiodynamic polarization to determine critical corrosion parameters, including corrosion potential and current density, along with electrochemical impedance spectroscopy to assess the stability and protective qualities of the oxide film. Surface analytical techniques provided detailed microstructural and compositional insights, with scanning electron microscopy revealing the morphology of corrosion products, energy-dispersive X-ray spectroscopy identifying elemental distribution in the passive layer, and X-ray diffraction confirming the chemical composition and crystalline structure of surface oxide. The results demonstrated distinct corrosion resistance behavior between the different processing conditions of the alloy. The laser powder bed fused (LPBF) specimens in the as-built condition exhibited superior corrosion resistance compared to their hot isostatically pressed (HIPed) counterparts, as evidenced by higher corrosion potentials and lower current densities. Microscopic examination revealed the formation of a dense, continuous layer of corrosion products on the alloy surface, indicating effective barrier protection against chloride ion penetration. A compositional analysis of all samples identified oxide film enriched with chromium, nickel, cobalt, aluminum, titanium, and silicon. XRD characterization confirmed the presence of chromium oxide (Cr_2_O_3_) as the primary protective phase, with additional oxides contributing to the stability of the film. This oxide mixture demonstrated the alloy’s ability to maintain passivity and effective repassivation following film breakdown.

## 1. Introduction

The performance of materials regarding strength and suitability for severe environments is essential for various industrial sectors, including aerospace, marine, military, and chemical industries. Historically, conventional materials like mild steel have not satisfied the requisite strength and corrosion resistance criteria in high-temperature applications [1,2]. In response to these issues, advanced alloys, including high-entropy alloys (HEAs) and nickel-based superalloys, have been developed to address the deficiencies, since they exhibit superior corrosion resistance and enhanced creep and fatigue characteristics at elevated temperatures [3]. HEAs and nickel-based superalloys are utilized in high-temperature environments, rendering them appropriate for gas turbines, boilers, and aircraft engine applications [4].

Metallic materials are particularly vulnerable to deterioration caused by ambient pollutants and low-quality fuels, including sulfur, salt, and chlorine [5]. The repeated heating and cooling of the materials induces changes in the microstructure, significantly compromising the material’s integrity [6]. Reports have shown that nickel-based superalloys have the ability to resist corrosion in these environments. A study by Osoba et al. [7] on Haynes 282 and Inconel 718 in hydrochloric acid showed that the Haynes material had an improved corrosion resistance over the Inconel 718 as a result of a more compact passive film over the surface and a larger volume fraction of chromium and molybdenum in this alloy. Since materials for engineering applications undergo different forms of deformation, which affects the crystal orientation of the material, it is important to understand the effect of crystallographic orientation on the corrosion of engineering materials. Yang et al. [8] studied the effect of crystallographic orientation in a single-crystal nickel superalloy in 3.5 wt.% NaCl. They reported an increase in corrosion rate in this order for the planes studied (001) < (111) < (011), and the lower corrosion rate of the (001) plane when compared to other planes was attributed to higher equivalent atomic packing density and more compact corrosion products within the plane. Similarly, Liu et al. [4] attributed the differences in the composition of the passive film to different nickel-based superalloys with different microstructures. The alloys tested were polycrystalline (cast alloy), nanocrystalline (NC), and single crystalline (SC (200)), in which the NC had better resistance to corrosion as the presence of titanium oxide and chromium (III) oxide improves the corrosion resistance of the NC alloys.

HEAs have also shown tremendous corrosion resistance, as they tend to form protective oxides [9]. However, due to the multiple composition of elements in the alloy, they are prone to galvanic corrosion [10]. Zang et al. [9] reported pitting corrosion in CrFeNi_2_ and CoCrFeNi_2_ alloys with a single FCC phase structure. It was also reported that CrFeNi_2_ is superior to CoCrFeNi_2_ due to the higher chromium content in the alloy. However, galvanic corrosion was reported in the AlCoCrFeNi_2_ alloy. According to Fu et al. [10], HEAs have been reported to perform better than 304 stainless steel, as the enrichment of chromium (III) oxide in the HEAs improves the resistance to corrosion [11]. Shang et al. [12] reported an order of defectiveness of FeO > CoO > NiO > Cr_2_O_3_ in chloride ions during pitting corrosion for HEAs. They reported that chromium plays a significant role in the corrosion resistance of FCC HEAs, not the mixture entropy. Though there had been a traditional notion that an alloy mixed with multiple elements could contain inclusions, precipitates, and segregation, which can result in pitting corrosion, the high entropy effect results in crystal structures such as HCP, FCC, and BCC. These structures reduce the possibility of segregation and galvanic corrosion [13].

The material used in this study is an oxide dispersion-strengthened (ODS) superalloy developed by the NASA Glenn Research Center for Extreme Temperature above 810 °C (GRX-810) by using NiCoCr produced via the laser powder bed fusion (LPBF) additive manufacturing technique. Y_2_O_3_ powder was used as a dispersoid to strengthen the alloy. Results reported by Smith et al. [14] showed that the NiCoCr alloy doped with Y_2_O_3_ performed better than the non-oxide dispersion-strengthened alloy sample of NiCoCr at a high temperature of 1093 °C in terms of tensile strength and twofold better in terms of ductility, indicating its suitability for high-temperature applications. Furthermore, GRX-810 was compared with other nickel-based superalloys, such as the wrought Inconel 718, Haynes 230, and Inconel 625, and results showed improved tensile strength. The as-built GRX-810 also showed tremendous strength of up to 1.3 GPa in cryogenic conditions (−196 °C) due to the FCC-to-HCP phase transformation [15].

GRX-810 has shown an improvement in strength and ductility compared to other nickel-based superalloys at elevated and cryogenic temperatures and has found applications in several engineering fields, such as aerospace, especially for use in turbine blade materials. It is therefore important to understand the corrosion behavior of the material, as its use in industrial environments means it is exposed to fuels and atmospheric air, which contains impurities such as Cl, S, and Na [5]. Hence, this research examines the corrosion behavior of the GRX-810 alloy in 3.5 wt.% NaCl, as there are no corrosion studies on the GRX-810 material in the literature yet. The electrochemical properties of the alloy were characterized using potentiodynamic polarization and electrochemical impedance spectroscopy (EIS). The alloy’s morphology was examined using scanning electron microscopy (SEM) with Electron Dispersive Spectroscopy (EDS), and X-ray diffraction (XRD) analysis was used to characterize the oxide film formed on the alloy’s surface.

## 2. Materials and Methods

The chemical composition of the as-built GRX-810, received from NASA (Cleveland, OH, USA), is 33% Co, 29% Cr, 1.5% Re, 0.3% Al, 0.25% Ti, 0.75% Nb, 3% W, and 0.05% C, and the remainder is Ni. The alloy and manufacturing processes are the same as those of Smith et al. [14]. The alloy was manufactured using the laser powder bed fusion additive manufacturing technique (AM-LPBF) using a scanning speed of 1000 mm/s, a laser power of 270 W, a hatch spacing of 80 µm, and a layer thickness of 40 µm to build cylindrical testing coupons. Powders of an average diameter of 10–53 µm and nanoscale Y_2_O_3_ were used as the dispersoid. Some materials were also hot isostatically pressed (HIPed) at 1185 °C and a pressure of 172 MPa for 4 h. In this research, the AM-LPBF samples were labeled “AB” while those that had been HIPed were labeled as “HIP”.

**Figure 1:**
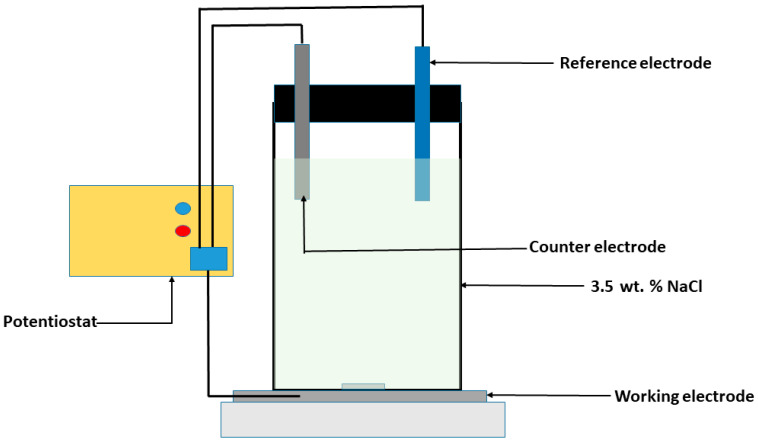
Schematics of the electrochemical cell setup.

Samples of 1.3 cm diameter and 3 mm thickness were cut for corrosion studies from each category of samples. The samples were hot-mounted on epoxy resin, and metallographically prepared using SiC grinding papers (#320–#1200) for polishing. The corrosion studies used the Gamry potentiostat 600+ (Warminster, PA, USA). The counter electrode is made of graphite rod, the reference electrode is the saturated calomel electrode (SCE), and the working electrode is the GRX-810 covered with an electrochemical mask with an exposed surface area of 1 cm^2^ along the build direction, as shown in Figure 1. The corrosion potential (E_corr_), current density (I_corr_), and the pitting potential (E_pit_) for the potentiodynamic analysis were determined using Gamry’s Echem analyst software (version 7.1.0). The electrochemical impedance spectroscopy (EIS) test results were also analyzed using Zview software (version 4.0h). A frequency range of 10^5^–10^−2^ Hz was used for the EIS measurements at an amplitude of 10 mV. The samples were stabilized in the 3.5 wt.% NaCl solution for 30 min prior to each round of electrochemical testing to allow for open circuit potential (OCP) stabilization.

The SEM images prior to and after corrosion tests were captured using the HITACHI SU-70 (Tokyo, Japan) equipped with an EDS detector at 20 kV. The XRD analysis was carried out using a Bruker D8 Venture Duo single crystal diffractometer (Billerica, MA, USA) with a Cu-Kα anode X-ray source, which had a wavelength of 1.5406 nm.

## 3. Results

### 3.1. Potentiodynamic Polarization

**Figure 2:**
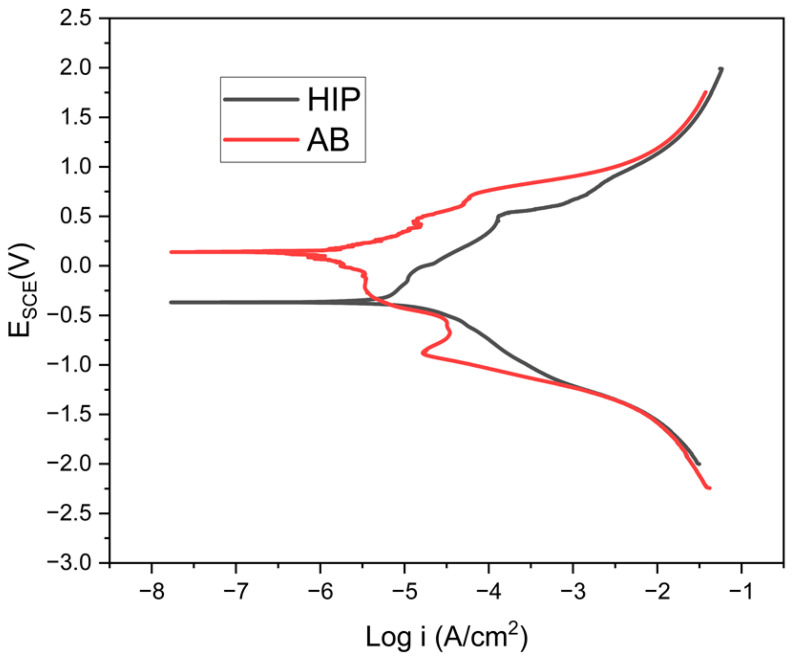
Potentiodynamic polarization curves of the GRX-810 sample for the as-built (AB) and hot isostatic pressed (HIP) conditions.

The potentiodynamic polarization curves in Figure 2 consist of the active–passive and transpassive regions, which suggest the formation of passive films at the corrosion potential, as Yanqui et al. [8] observed for nickel-based superalloys. The corrosion potential indicates the corrosion reaction; hence, the higher the Ecorr value, the better the corrosion resistance, and vice versa for the corrosion current density [16,17]. The Ecorr of the AB had the most resistance to corrosion, at 187 mV, compared to the HIP sample at −406 mV, as presented in Table 1. Similarly, the corresponding Icorr was at 1.8 and 11 µA/cm^2^, respectively. The results show that the HIP sample will corrode faster than that of the AB, as the HIPing process results in an increased grain boundary, which is usually the site for aggressive corrosion attack in materials [18,19]. Furthermore, the HIPing process is also a thermomechanical process involving some grain deformation. Thus, this weakens the adhesion between the passive film and the matrix and further prevents the passive film’s reformation on the alloy’s surface [20]. The Epit of the AB sample is higher than that of the HIP sample, showing less susceptibility of the AB sample compared to the HIP sample.

### 3.2. Electrochemical Impedance Spectroscopy (EIS)

**Figure 3:**
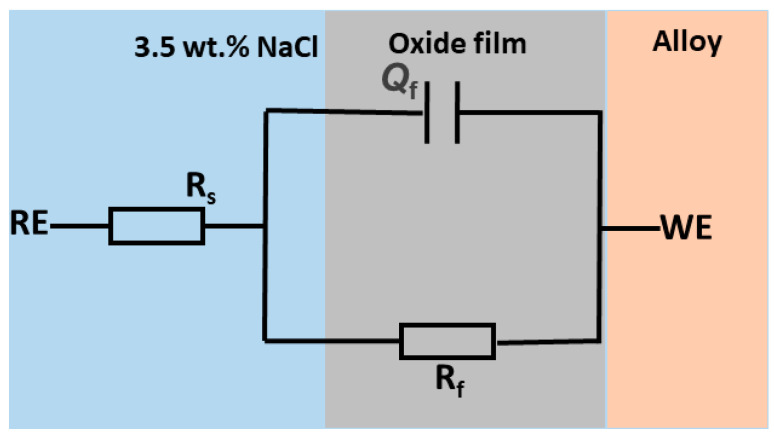
Equivalent circuit of GRX-810 alloy.

The EIS test measures the resistance (impedance) to the flow of alternating current at various frequencies [21]. The electrochemical cell analysis results include the solution resistance (Rs), constant phase element (Q_f_) of oxide film, and film resistance (R_f_), which are presented in Table 2, and the equivalent circuit for the alloy, as shown in Figure 3. It is important to note that the chi-squared error is less than 10^−3^, signifying the accuracy of the fitted model. Furthermore, the variation in the R_s_ (16.13–19.16 Ωcm^2^) is small, showing the stability of the solution used in the cell.

**Figure 4:**
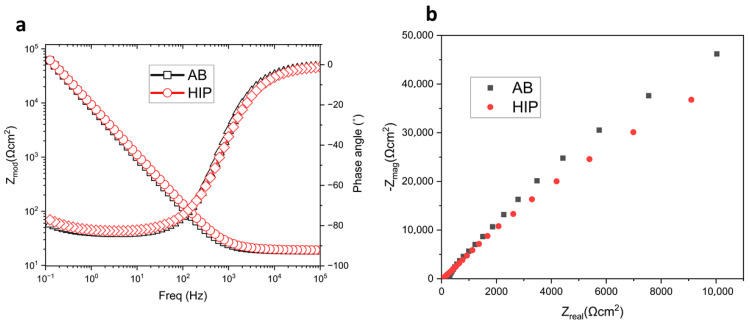
Chart of (**a**) Bode plot of GRX-810 alloy; (**b**) Nyquist plot of GRX-810 alloy.

The Bode and Nyquist plots shown in Figure 4a,b show the behavior of the passive film formed on the surface of the alloys. The Nyquist plot displays various arcs of differing radii, with the arc for the AB sample being larger than that of the HIP sample, aligning with the observed trend in charge transfer resistance (R_f_) [22,23]. This suggests that the EIS test process was governed by charge transfer within the single electric layer on the alloy’s surface, with the phase angle of the two samples close to 90°, indicating the capacitive behavior of the alloys [18]. A reduced R_f_ indicates increased active sites for corrosion inside the passive film. The greater the radius, the more enhanced the stability of the passive film, illustrating that the AB sample exhibits enhanced film formation stability compared to the HIP sample.

From the fitted electrical circuit shown in Figure 3, the impedance can be represented as

(1) $$Z=\frac{1}{Y_{0}}({j\omega )}^{-n}$$

where *Y_*0*_* is the CPE amplitude, *n* is the depression coefficient (0 < *n* < 1), j is an imaginary unit, and *ω* is the angular frequency.

The AB has a lower Q_f_ amplitude compared to the HIPed sample, as shown in Table 2, further demonstrating the compactness of the passive film compared to the HIP sample. A depression coefficient close to 1 is said to demonstrate general stability, showing that the Q_f_ is close to an ideal capacitor. The AB sample had a value of 0.94, which is comparatively higher than the HIPed of 0.73. Works in the literature have also associated higher depression values with smoother electrode surfaces [24]. Also, the R_f_ value, which is the resistance of the oxide film, is higher in the AB alloy, indicating an improved resistance to corrosion (highly stable film formed) compared to the HIP sample.

### 3.3. Morphology of GRX-810

**Figure 5:**
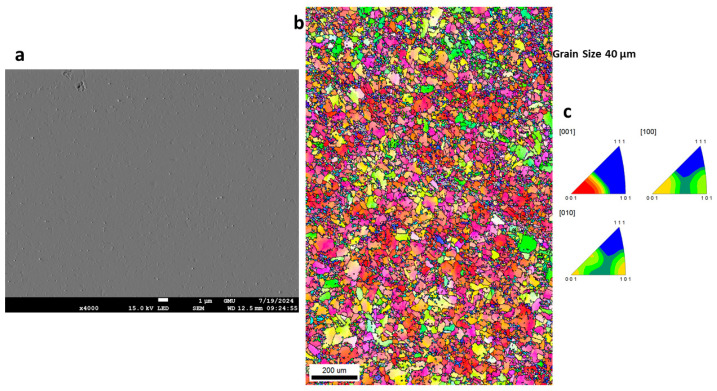
SEM images of as-built GRX-810 prior to corrosion: (**a**) surface morphology; (**b**) inverse pole figure (IPF) image; (**c**) IPF plot.

**Figure 6:**
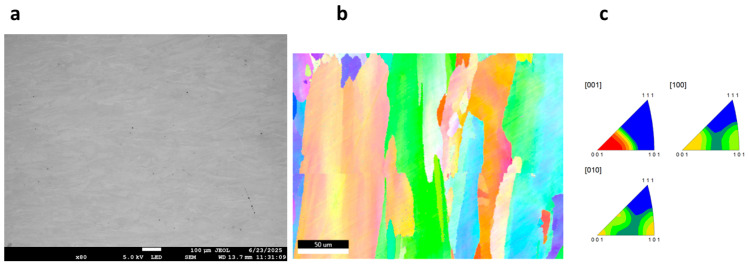
SEM images of HIPed GRX-810 prior to corrosion: (**a**) surface morphology; (**b**) inverse pole figure (IPF) image; (**c**) IPF plot.

**Figure 7:**
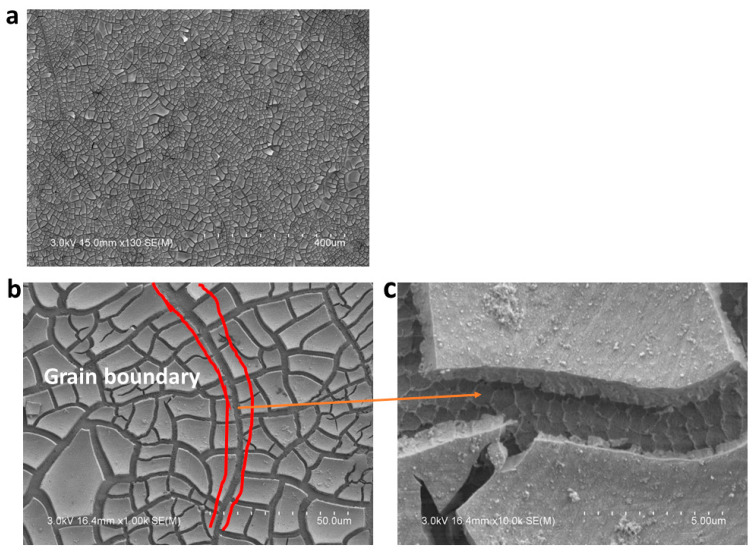
Morphology of the AB GRX-810 after corrosion: (**a**) lower magnification showing the corrosion products on the surface of the alloy; (**b**) spalling around the grain boundary; (**c**) higher magnification around the grain boundary.

**Figure 8:**
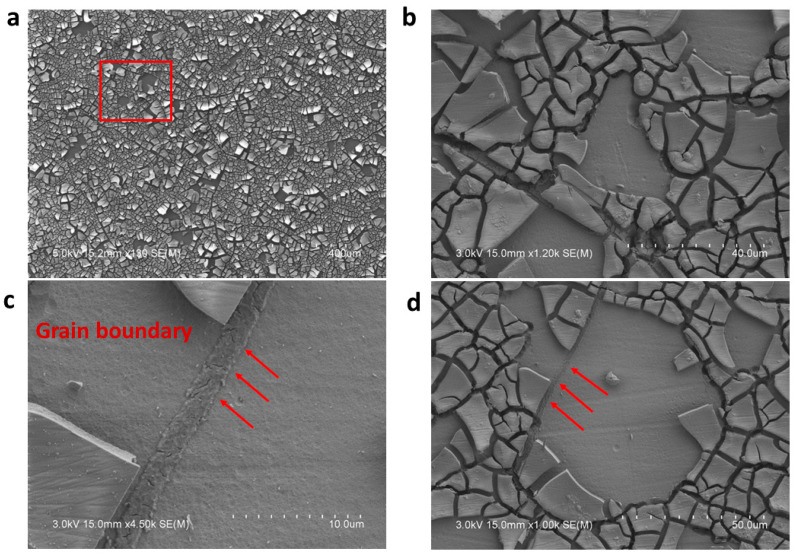
Micrograph of the HIP GRX-810 after corrosion, showing (**a**) the corrosion product on the surface of the alloy; (**b**) higher magnification showing spalling on the surface of the alloy; (**c**) the corrosion product around the grain boundary; and (**d**) spalling around the grain boundary.

The surface morphology and electron backscatter diffraction (EBSD) images of the as-built and HIPed alloy prior to corrosion are shown in Figure 5a–c and Figure 6a–c. It can be observed that the surfaces are nearly defect-free, as a relative density of 99.96% was reported for the optimized print parameters [14]. The IPF image of the as-built samples shows the grains to be equiaxed and fine (~40 µm), while that of the HIPed samples shows coarse and increased grain sizes of ~193 µm, as a result of the thermomechanical process of HIPing that has occurred in the material. To further understand the corrosion behavior of the materials, the morphology of the alloy’s surface was examined using SEM, followed by EDS analysis. Figure 7a shows the corrosion products, which are formed on the surface of the alloy during electrochemical testing (EIS and Potentiodynamic test) and are mainly salt (NaCl), on the surface of the GRX-810 as-built alloy, which is observed to be denser than that of the HIPed GRX-810 alloy. Some cracks in the corrosion product can be seen, which could be a result of spalling, with higher magnifications of the micrograph shown in Figure 7b,c, revealing adhesion of the products around the grain boundaries, which might be a result of the grain boundaries being unable to protect the alloy in the passive state. Similarly, the HIPed GRX-810 sample showed spalling on the surface of the alloy and had a less dense corrosion product on the surface of the alloy when compared to that of the AB sample, as shown in Figure 8a–d and visualized with the red box and arrows in Figure 8a,c,d.

**Figure 9:**
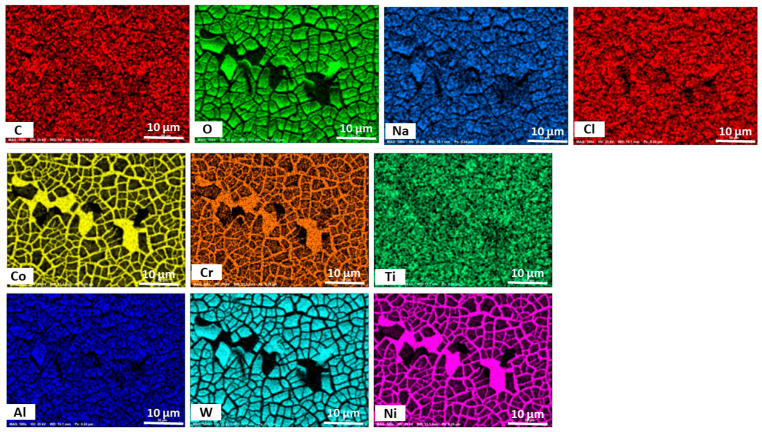
Electron dispersive spectroscopy mapping of the elemental composition of the AB GRX-810 alloy after corrosion.

**Figure 10:**
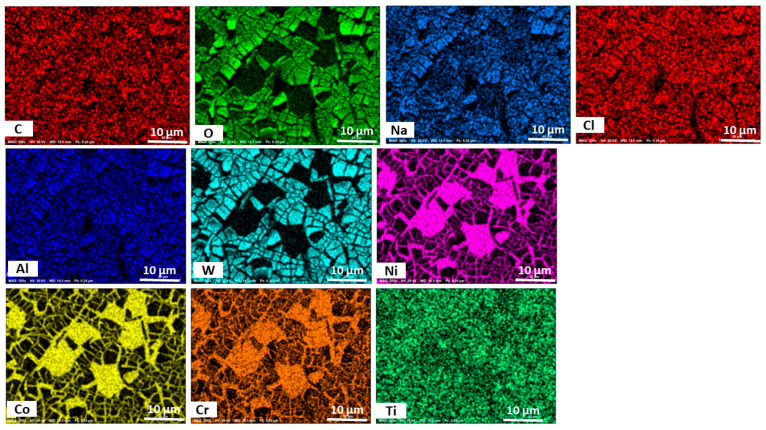
Electron dispersive spectroscopy mapping of the elemental composition of the HIP GRX-810 alloy after corrosion.

To obtain further information on the elemental composition of the surface of the alloys, they were characterized by randomly selecting the profile and the EDS results, as shown in Figure 9 for the AB GRX-810 and Figure 10 for that of the HIP GRX-810. The images show the presence of oxygen on the surface of the metal, indicating the formation of oxide films on the surface. Elements detected on the surface of the alloys include Ni, Co, Cr, Al, W, C, and Ti, indicating the possibility of the formation of CoCr_2_O_4_, NiCr_2_O_4_, and TiO_2_, which are beneficial to the resistance of corrosion in these alloys, as these oxides inhibit corrosion [22,23,25]. The energy spectrum for all samples are provided in Supplementary File S1 for the AB sample and S2 for the HPed sample respectively.

### 3.4. Oxide Film Formation

#### XRD Analysis

**Figure 11:**
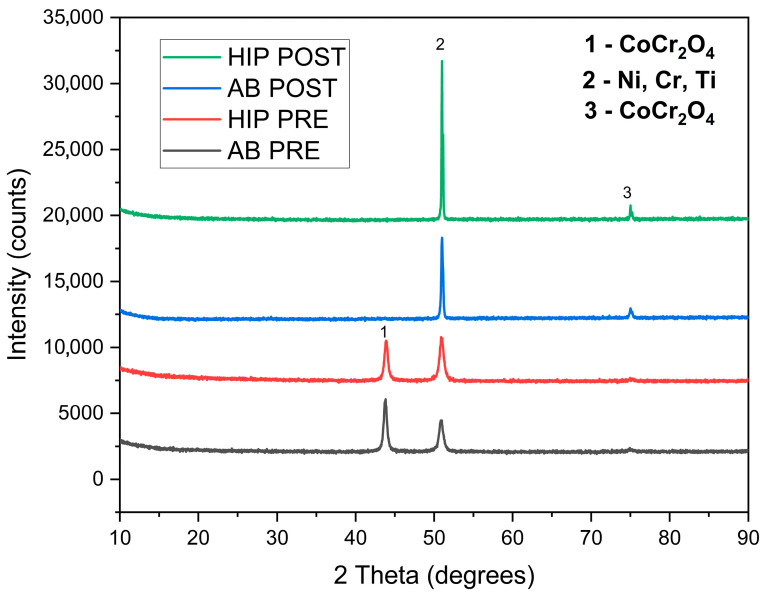
XRD pattern of the AB and HIP GRX-810 before and after the corrosion test.

Figure 11 shows the XRD pattern of the GRX-810 alloy before and after corrosion tests. Before corrosion, the alloy showed peaks of (111) at 43.82°, (200) at 50.98°, and (220) at 74.93°, which are typical of FCC structures such as nickel-based superalloys and suggest γ and γ^’^ phases [18,26]. The post-corrosion XRD analysis of the surface of the samples revealed peaks that corresponded to the presence of oxides CoCr_2_O_4_ (1,3) and NiCr_2_O_4_ (1,3) and those of Ni, Ti, and Cr (2). However, there is a disappearance of the (111) peak, which could be attributed to the anisotropic nature of corrosion reactions on the surface of the alloy and also the dissolution of unstable phases such as γ^’^ precipitates to form new phases such as NiO and Cr_2_O_3_, which could overshadow the (111) peak [27,28]. Furthermore, corrosion products are rich in metal oxides, and they form an amorphous layer on the surface of the alloy, which can further overshadow the existing peaks [29]. The (220) peaks also show a slight increase in intensity in the post-corrosion XRD analysis when compared to that of the pre-corrosion samples for both AB and HIP conditions as a result of the increase in oxide formation on the passive film formed on the surface of the alloy [10].

## 4. Discussion

This study was carried out to understand the corrosion mechanism of the GRX-810 alloy with Ni, Co, and Cr as the matrix’s major elements. GRX-810 is an MEA with an FCC structure. The alloy was processed using the LPBF additive manufacturing technique, and some of the samples were further processed through the HIP technique to improve the density of the alloy and close up the pores that might have been present during the manufacturing process, which could contribute to the behavior of the materials under a corrosive environment. All samples were tested for corrosion in 3.5 wt.% NaCl and were characterized using potentiodynamic polarization, EIS, SEM/EDS, and XRD.

Potentiodynamic polarization tests were carried out on all samples, and the E_corr_, I_corr_, and E_pit_ values were used to characterize the corrosion rate. The performances of all the GRX-810 samples were in the order of AB > HIP with decreasing E_corr_ value. The results were further corroborated by the EIS analysis, which also agreed with the potentiodynamic polarization tests. The surface morphology of all the samples shows adhesion and low spalling of the corrosion products, which shows the tendency of materials to form passive film on the surface of the alloy.

**Figure 12:**
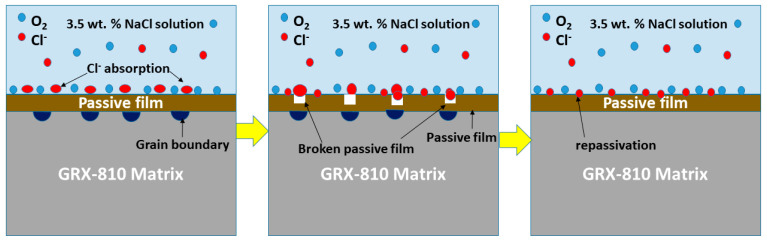
Schematics of the corrosion mechanism of GRX-810 in 3.5 wt.% NaCl.

Though pits were not observed on the surfaces of the alloys, the corrosion mechanism starts with the initial oxide layer formed on the alloy surface in the air, which acts as a diffusion barrier, slowing down corrosion and oxidation. The oxide film is destroyed when it is attacked by Cl^−^ in a simulated marine environment, resulting in the displacement of O^2−^ and OH^−^ and penetration of grain boundaries and defects on the surface of the alloy. However, an oxide film formed by repassivation further inhibits corrosion attacks. In GRX-810, the Cr composition is above 25%, suggesting rapid repassivation forming a Cr_2_O_3_-rich protective film [17]. The schematic of the corrosion mechanism is shown in Figure 12.

The study by Liu et al. [4] shows that smaller grain sizes ensure the rapid reformation of oxide layers, resulting in a more compact passive film. This corroborates the results of this work, where the AB has large numbers of smaller grain sizes, thereby improving the corrosion resistance of the AB sample over the HIPed. The HIPed exhibited a lower corrosion resistance as a result of grain growth that emanated from the HIPing process, hence reducing the corrosion ability of the GRX-810 HIPed alloy. Additionally, from post-corrosion XRD patterns of both the AB and HIP samples, there is a suppression of the (111) peak, which suggests preferential corrosion along crystallographic planes, possibly due to the breakdown of γ’ precipitates and surface passivation by Cr, Ni, and Ti-rich oxides, as discussed by Gradl et al. [15] in their research investigation, where the presence of Nb and Ti-rich MC carbides were seen along the grain boundaries of the GRX-810.

Another prevailing theory that supports the alloys’ corrosion behavior is that these alloys are FCC metals and possess high surface atomic density to a great degree, which reduces diffusion paths for Cl^−^, as described by Yanqui et al. [6]. Furthermore, the presence of Cr in the matrix of the samples could result in the formation of a Cr_2_O_3_ layer, which is known to inhibit corrosion [22]. Also, from the standard Gibbs free energies of the formation of oxides, Cr_2_O_3_ < CoO < NiO, ascertaining that Cr has the highest binding energy capability. Thus, the Cr_2_O_3_ formed a passive film over the surface of the alloy to a significant degree [10,25]. However, the formation of other oxides, such as CoCr_2_O_4_ and NiCr_2_O_4_, has not been ruled out, as these oxides were detected in the XRD analysis carried out. Furthermore, grain coarsening in the materials that had undergone thermomechanical treatment, such as HIP GRX-810, could result in reduced film adhesion and increased I_corr_ when compared to the AB GRX-810, which has finer grains [30].

## 5. Conclusions

This study examined the corrosion behavior of GRX-810 manufactured via the LPBF additive manufacturing technique in AB and HIP conditions and compared the corrosion properties in 3.5 wt.% NaCl. The following conclusions were deduced from the research.

1. Based on the potentiodynamic results, the corrosion resistance rate is as follows: AB GRX-810 > HIP GRX-810. This shows decreasing Ecorr value and relative stability in marine environments. The passive film formed after repassivation was observed to be more compact in the AB GRX-810 sample compared to the HIP sample.
2. Analysis of the EIS data suggested that the passive film formed on the GRX-810 alloy can be modeled as a single-layer structure, indicative of a homogenous and compact barrier layer. The high impedance values and phase angle behavior further imply that the alloy surface is densely covered with corrosion products, which may enhance the protective characteristics of the oxide film by reducing ion transport and slowing down corrosion kinetics.
3. The SEM/EDS results suggest the possibility of the formation of oxide films of Cr, Ni, Co, and Ti for the GRX-810 alloy, with the XRD results confirming the formation of Cr_2_O_3_ on the surface of the alloy after polarization. However, future studies employing surface-sensitive techniques like XPS would be valuable to gain deeper insight into the atomic-level structure and composition of the oxide layer formed on the surface of the alloy.

## Figures and Tables

**Table 1:** Potentiodynamic polarization parameters of GRX-810 alloy.

Specimen	E_corr_ (mV_sce_)	I_corr_ (µA/cm^2^)	E_pit_ (mV_sce_)
GRX-810-AB	187	1.80	505
GRX-810-HIP	−406	11	437

**Table 2:** Parameters of the fitting of the equivalent circuit for EIS results of GRX-810 alloy.

Specimen	R_s_ (Ωcm^2^)	Q_f_ (Ω^−1^/cm^2^)	R_f_ (Ωcm^2^)	n	Х^2^
GRX-810 AB	19.16	2.5 × 10^−5^	4.8 × 10^11^	0.94	6.14 × 10^−4^
GRX-810 HIP	16.13	1.11 × 10^−4^	1.4 × 10^4^	0.73	7.9 × 10^−3^

## Data Availability

The raw data supporting the conclusions of this article will be made available by the authors on request.

## Supplementary Materials

The following supporting information can be downloaded at: https://www.mdpi.com/article/10.3390/ma18143252/s1, Supplementary File S1: energy spectrum of sample AB; Supplementary File S2: energy spectrum of sample HIP.
