# Supplementary material for: Corrosion Behavior of Additively Manufactured GRX-810 Alloy in 3.5 wt.% NaCl
Source: Materials (Basel). 2025 Jul 10;18(14):3252. doi: 10.3390/ma18143252 (PMC12298897; doi:10.3390/ma18143252)
Supplement: Supplementary file 1 [file materials-18-03252-s001.zip › S1. Map_EDS_AB.pdf]

Application Note

Company / Department

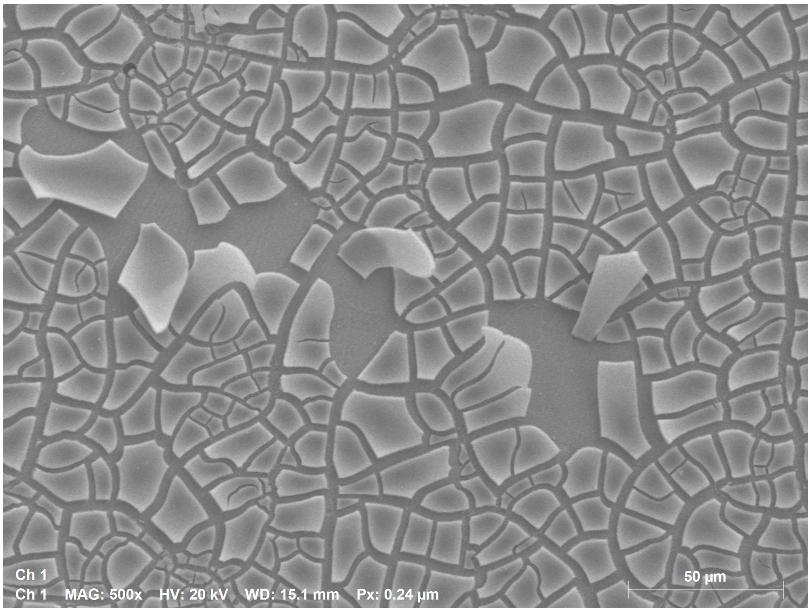

Name	Date	Time	HV [kV]	Mag	WD [mm]
Ch 1	2/27/2025	10:33:34 AM	20.0 keV	500x	15.1 mm

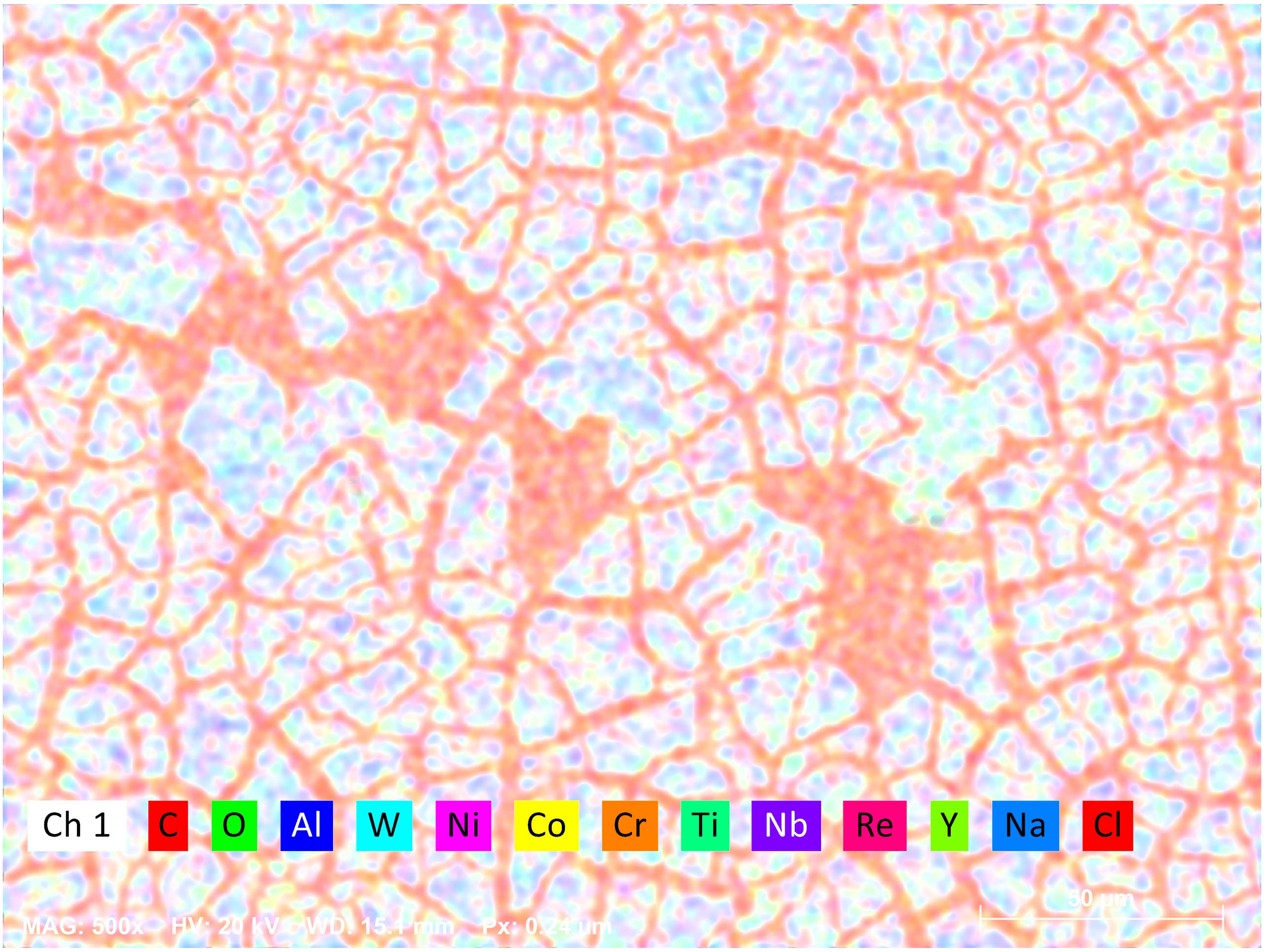

Date	Time	HV [kV]	Mag	WD [mm]
2/27/2025	10:33:42 AM	20.0 keV	500x	15.1 mm

BRUKER

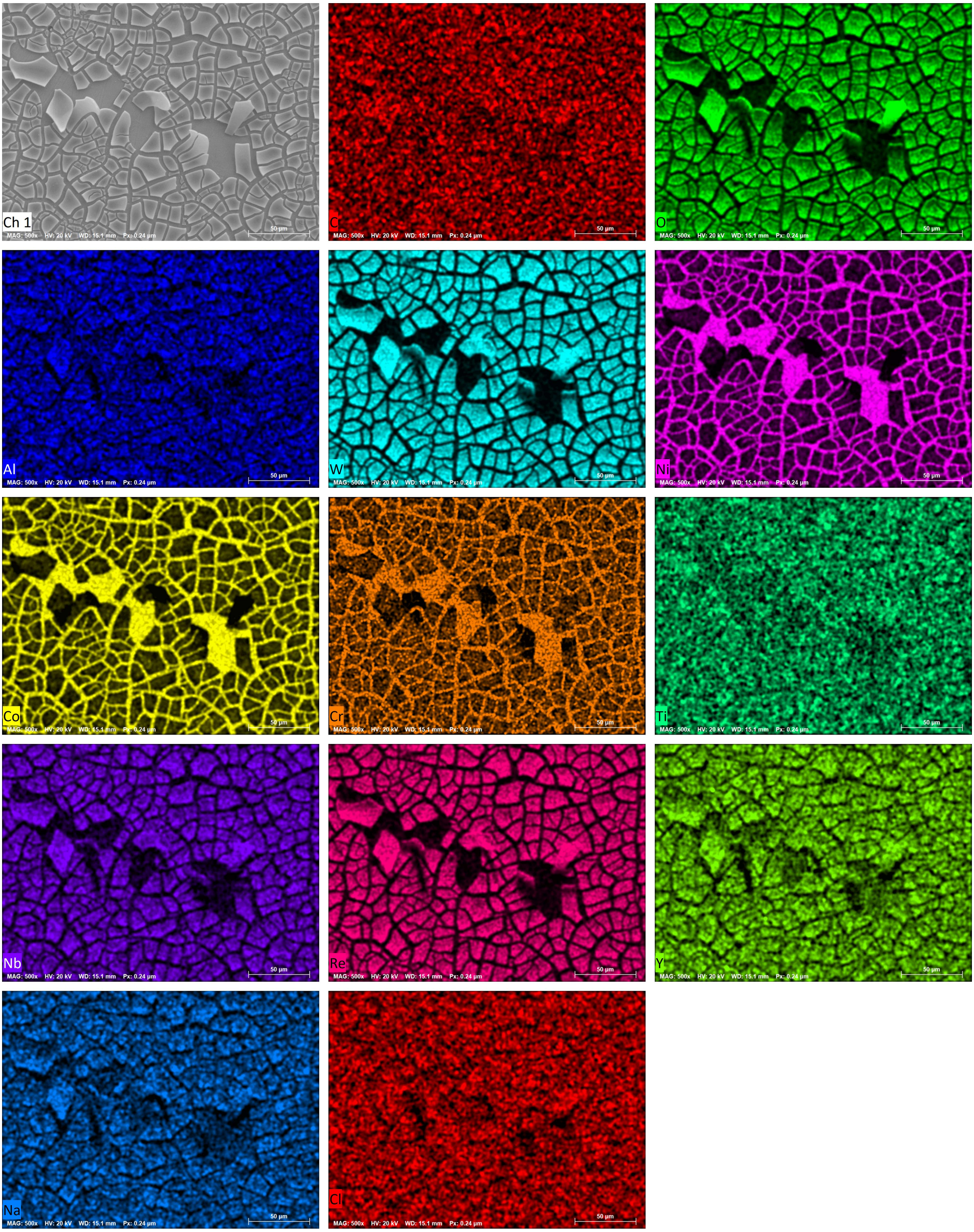

Date	Time	HV [kV]	Mag	WD [mm]
2/27/2025	10:33:42 AM	20.0 keV	500x	15.1 mm

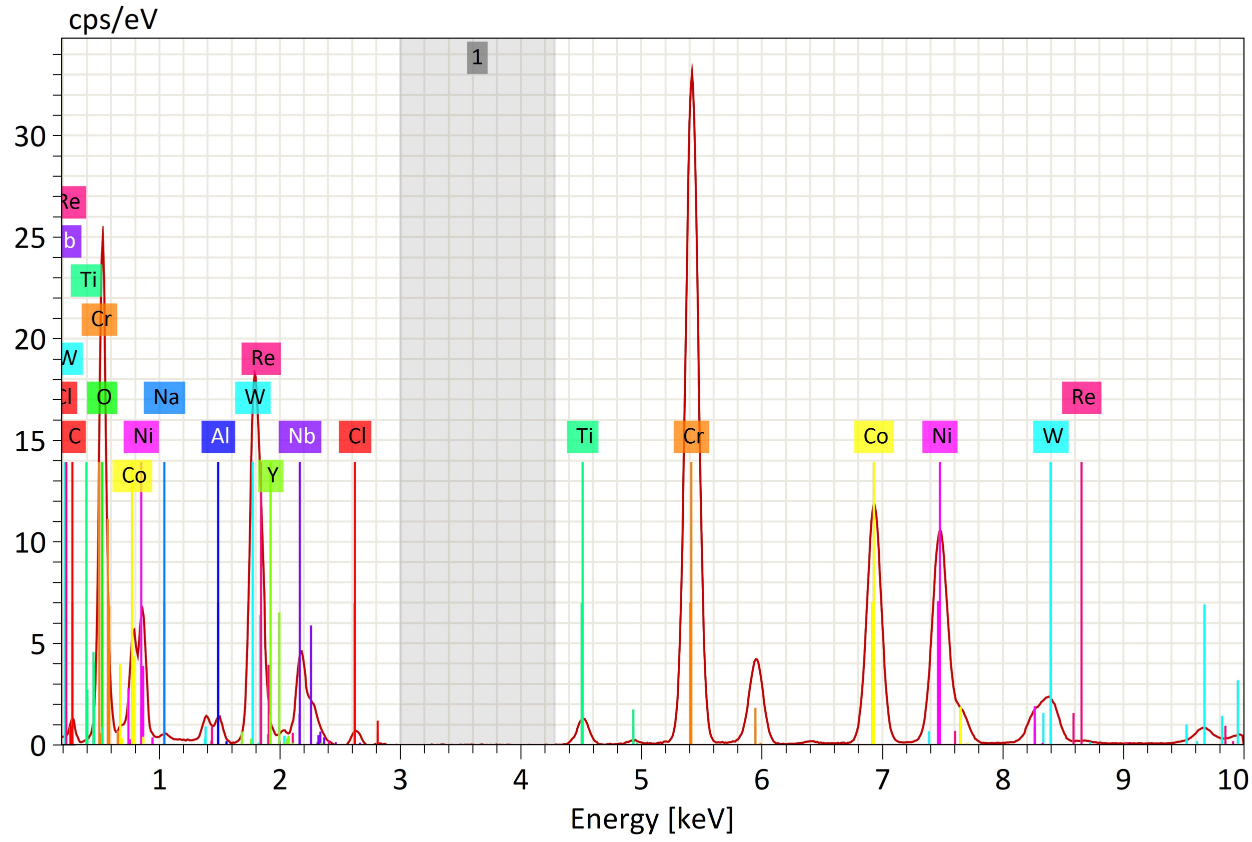

Map

Element	At. No.	Netto	Mass [%]	Mass Norm. [%]	Atom [%]	abs. error [%] (1 sigma)	rel. error [%] (1 sigma)
Carbon	6	25950	3.04	2.77	9.04	0.41	13.61
Oxygen	8	453348	18.66	16.99	41.64	2.01	10.79
Aluminium	13	40136	0.54	0.49	0.71	0.05	9.59
Tungsten	74	209480	15.26	13.89	2.96	0.44	2.91
Nickel	28	639988	19.54	17.79	11.88	0.54	2.78
Cobalt	27	694135	19.50	17.75	11.81	0.54	2.78
Chromium	24	1732670	27.19	24.75	18.66	0.75	2.77
Titanium	22	60490	0.81	0.74	0.61	0.05	5.95
Niobium	41	233389	3.89	3.54	1.49	0.17	4.32
Rhenium	75	6972	0.56	0.51	0.11	0.05	9.43
Yttrium	39	4751	0.09	0.08	0.04	0.04	40.74
Sodium	11	16785	0.52	0.47	0.81	0.06	11.68
Chlorine	17	24400	0.25	0.23	0.25	0.03	13.96
		**Sum**	**109.84**	**100.00**	**100.00**		
