# Supplementary material for: Corrosion Behavior of Additively Manufactured GRX-810 Alloy in 3.5 wt.% NaCl
Source: Materials (Basel). 2025 Jul 10;18(14):3252. doi: 10.3390/ma18143252 (PMC12298897; doi:10.3390/ma18143252)
Supplement: Supplementary file 1 [file materials-18-03252-s001.zip › S2. GRX810 HIP EDS.pdf]

Application Note

Company / Department

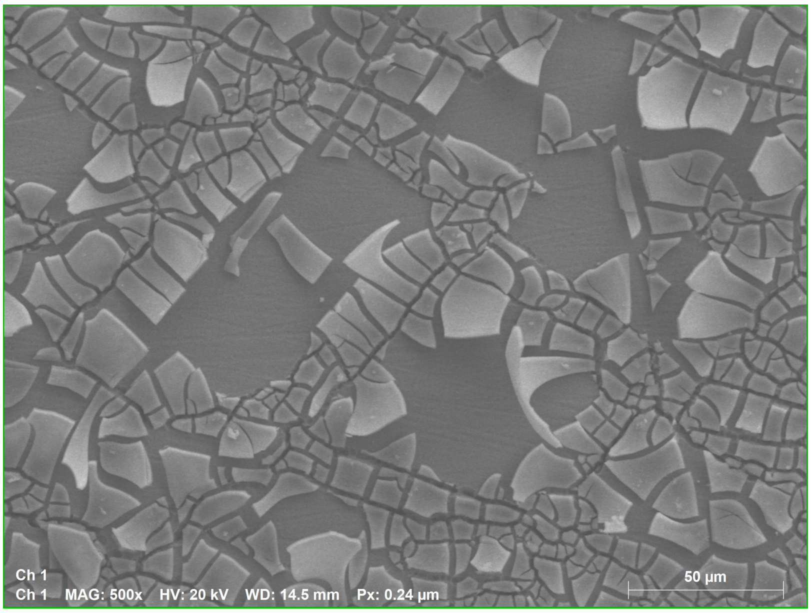

Name	Date	Time	HV [kV]	Mag	WD [mm]
Ch 1	2/27/2025	10:56:03 AM	20.0 keV	500x	14.5 mm

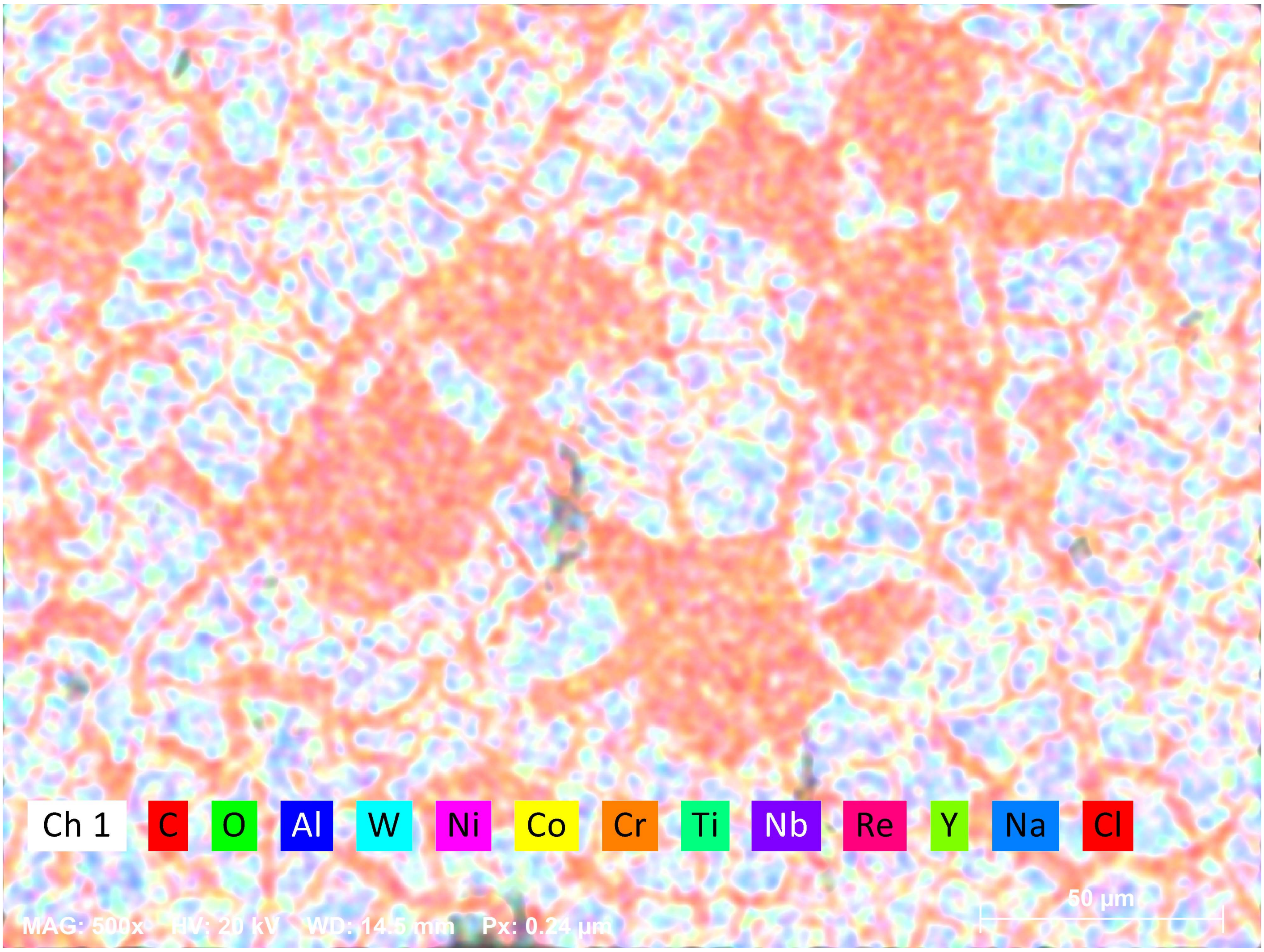

Date	Time	HV [kV]	Mag	WD [mm]
2/27/2025	10:56:14 AM	20.0 keV	500x	14.5 mm

BRUKER

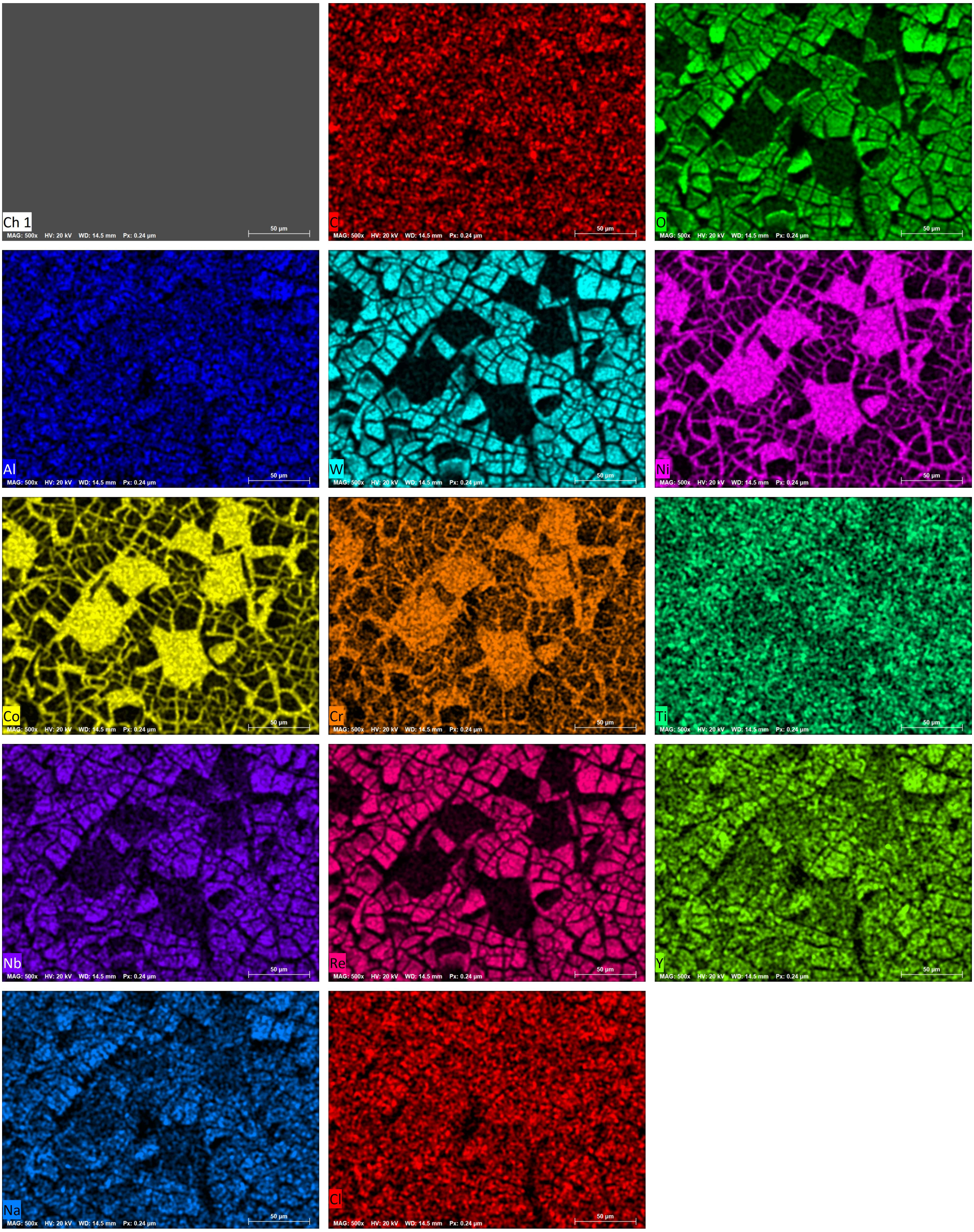

Date	Time	HV [kV]	Mag	WD [mm]
2/27/2025	10:56:14 AM	20.0 keV	500x	14.5 mm

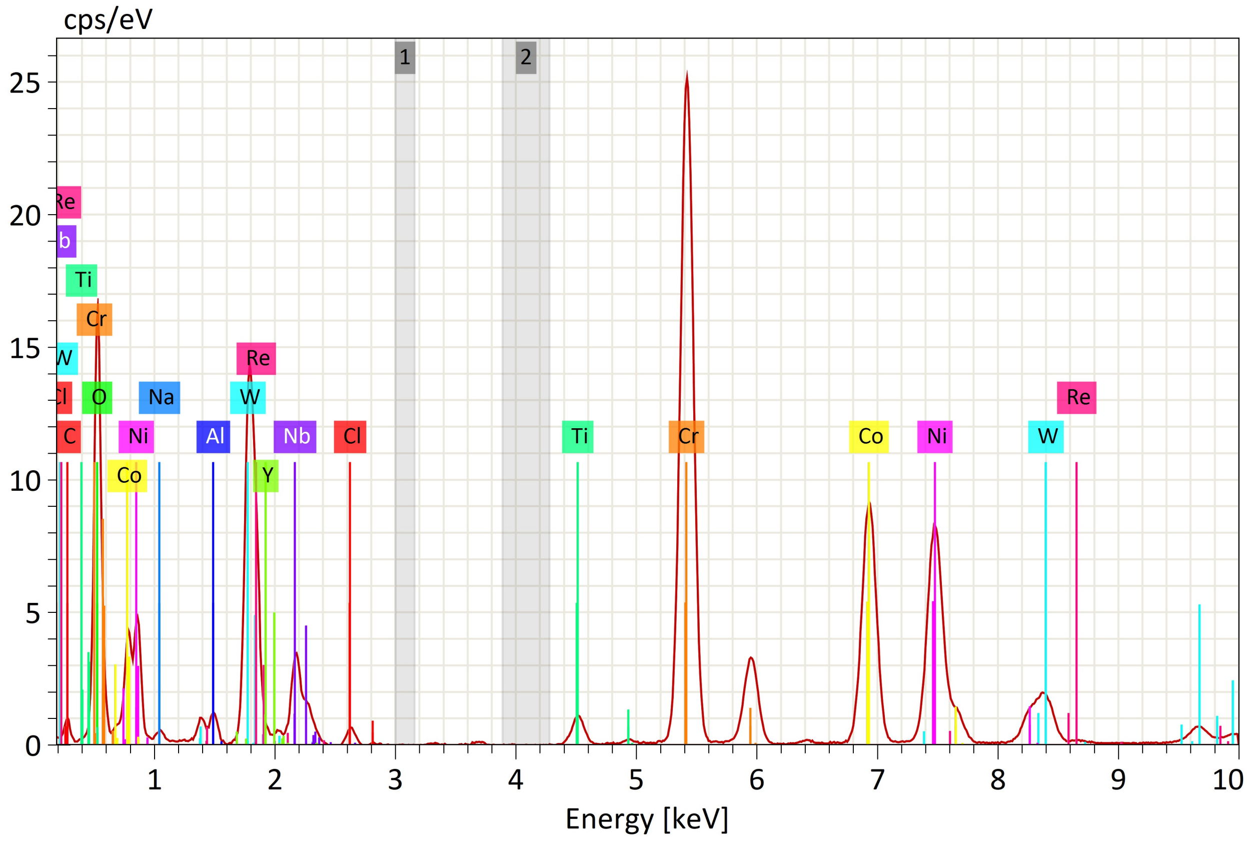

Map

Element	At. No.	Netto	Mass [%]	Mass Norm. [%]	Atom [%]	abs. error [%] (1 sigma)	rel. error [%] (1 sigma)
Carbon	6	11797	3.10	2.81	9.54	0.46	14.93
Oxygen	8	176873	16.50	14.97	38.13	1.85	11.20
Aluminium	13	20503	0.64	0.58	0.87	0.06	9.00
Tungsten	74	103551	16.52	14.99	3.32	0.48	2.93
Nickel	28	298858	19.97	18.11	12.58	0.56	2.79
Cobalt	27	319273	19.65	17.82	12.32	0.55	2.78
Chromium	24	791573	27.32	24.78	19.42	0.76	2.77
Titanium	22	30031	0.89	0.81	0.69	0.05	5.79
Niobium	41	103278	4.03	3.66	1.60	0.17	4.32
Rhenium	75	3307	0.58	0.53	0.12	0.06	10.80
Yttrium	39	1734	0.08	0.07	0.03	0.04	52.97
Sodium	11	9138	0.65	0.59	1.05	0.07	10.92
Chlorine	17	13405	0.32	0.29	0.33	0.04	11.95
		**Sum**	**110.26**	**100.00**	**100.00**		
